# Regional radiotherapy versus an axillary lymph node dissection after lumpectomy: a safe alternative for an axillary lymph node dissection in a clinically uninvolved axilla in breast cancer. A case control study with 10 years follow up

**DOI:** 10.1186/1748-717X-2-40

**Published:** 2007-10-30

**Authors:** Patty H Spruit, Sabine Siesling, Marloes AG Elferink, Ernest JA Vonk, Carel JM Hoekstra

**Affiliations:** 1 Radiotherapeutic Institute RISO, Deventer, The Netherlands; 2 Comprehensive Cancer Centre Stedendriehoek Twente, Enschede, The Netherlands

## Abstract

**Background:**

The standard treatment of the axilla in breast cancer used to be an axillary lymph node dissection. An axillary lymph node dissection is known to give substantial risks of morbidity. In recent years the sentinel node biopsy has become common practice. Future randomized study results will determine whether the expected decrease in morbidity can be proven.

**Methods:**

Before the introduction of the sentinel node biopsy, we conducted a study in which 180 women of 50 years and older with T1/T2 cN0 breast cancer were treated with breast conserving therapy. Instead of an axillary lymph node dissection regional radiotherapy was given in combination with tamoxifen (RT-group). The study group was compared with 341 patients, with the same patient and tumour characteristics, treated with an axillary lymph node dissection (S-group).

**Results:**

The treatment groups were comparable, except for age. The RT-group was significantly older than the S-group. The median follow up was 7.2 years. The regional relapse rates were low and equal in both treatment groups, 1.1% in RT-group versus 1.5% in S-group at 5 years. The overall survival was similar; the disease free survival was significant better in the RT-group.

**Conclusion:**

Regional recurrence rates after regional radiotherapy are very low and equal to an axillary lymphnode dissection.

## Background

Before the introduction of the sentinel node biopsy, the standard treatment of the axilla in early stage breast cancer was an axillary lymph node dissection (ALND). An ALND results in a high risk of morbidity, while in 70% of the postmenopausal patients with early stage breast cancer an ALND would be unnecessary because of the absence of lymph node metastases [1-4]. Lymph oedema of the arm (2–28%) [4-6] and shoulder function impairment (5–19%) [4] are most debilitating but even more patients suffer from dysesthesia and pain (23–31%) [6-8].

An alternative to an ALND is primary regional radiotherapy. This is expected to give less morbidity: arm oedema to a lesser extent (0–9%) and shoulder function impairment (0–1%) [4,9] are noted. Dysesthesia and pain are neither mentioned nor expected.

It is always a challenge to find alternative treatments leading to less complaints but this may not lead to major changes in tumour control and survival rates.

This study was initiated under the assumption that the control rates and survival after regional radiotherapy instead of an ALND in breast cancer patients of 50 years and older, are comparable while the morbidity is less, resulting in a better quality of life.

## Methods

### Patient population

Over the period 1991–2000 women aged 50 years and older with T1 or T2 breast cancer, with clinically negative axilla (cN = 0, assessed by physical examination) diagnosed in the Deventer Hospital were treated with axillary radiotherapy instead of an ALND. If the patients preferred the standard treatment (ALND), this was given. To compare the study group, a control group was compiled from all patients treated in the Gelre Hospitals Apeldoorn, over the same period with the same patient and tumour characteristics, and the patients from the Deventer Hospital not treated within the study protocol. Both patient groups were retrieved by using the regional cancer registry of the Comprehensive Cancer Centre Stedendriehoek Twente. In this population based cancer registry all newly diagnosed malignancies are registered. All charts were reviewed by the radiation oncologists of our department, concerning patient and tumour characteristics, recurrences and survival. All TNM stages were converted into the latest version (sixth edition) [10].

### Treatment

Patients treated with lumpectomy without an ALND (RT-group), were irradiated on the breast, the axillary, supra- and infraclaviculary lymph nodes and the ipsilateral internal mammary chain. The radiotherapy started within 6 weeks after surgery. All patients were irradiated on the breast with 2 tangential fields on a linear accelerator with 6 or 10 MV photon beams. Total dose to the entire breast was 50 Gy, in 2 Gy fractions, followed by a boost at the tumour bed of 14 Gy. Irradiation of the axilla was performed with the so-called McWirther technique to prevent overlap with the tangential fields of the breast: supraclavicular and axillar with two ventrodorsal opposed half-beam fields, 6 MV photon beams, the medial part only from ventral, specified at 3 cm. The internal mammary chain was irradiated with a single ventrodorsal beam, 26 Gy with a 6 MV photon energy beam followed by 24 Gy 12 MeV electrons. In individual cases the internal mammary chain was not irradiated as a result of the physician's opinion based on age of the patient, tumour characteristics (like size) or localisation of the tumour (lateral part of the breast).

Although the receptor status was unknown in most patients, adjuvant hormonal treatment, i.e. tamoxifen 20 mg a day, was given to all patients. The duration of the hormonal treatment was at least 2 years. Evolving data during the period of the current study showed the possible benefits of extended hormonal therapy use, therefore some patients were treated for 5 years.

Patients who underwent a lumpectomy and an ALND, a level 1 and 2 resection, (S-group) were irradiated on the breast, with 2 tangential fields as described above. Depending on the pathological findings of the tumour and the lymph nodes, regional radiotherapy and adjuvant therapy (hormonal and/or chemotherapy) were given according to the National guidelines. These patients were irradiated on the internal mammary lymph nodes in case of a positive axilla and/or a tumour in the medial part of the breast. When the EORTC trial investigating the role of irradiation of the internal mammary lymph nodes [11] was initiated, the patients were only irradiated on this chain within the participation of this trial (16 patients of the S-group participated in this trial).

### Follow-up

For all selected patients the follow-up was completed by performing a chart review.

All patients were followed every 3 months in the first year, every six months from the second to the tenth year, by the radiation oncologist alternated with the surgeon. With every visit the patient was checked for locoregional recurrence, signals for distant metastases and side effects. Unfortunately no validated morbidity lists were used so a reliable overview of differences in morbidity can not be given. Every year a mammography was made. After 10 years of disease free follow-up, the patients were discharged.

If information about follow-up was lacking in the patient file, general practitioners were contacted.

### Statistical analysis

Differences between the two treatment groups were determined using a chi-squared test. Disease free survival and overall survival were calculated, using multivariate Cox regression in SPSS (Statistical Package for Social Sciences). In the disease free survival analysis, the endpoint was the first local recurrence, regional recurrence or metastasis. All deaths, irrespective of cause of death, were taken as endpoint in the overall survival. Patients without an endpoint were censored at the end of their follow-up in the survival analysis. The effect of the treatment (RT or surgery) was adjusted for the variables tumour stage pT, age and irradiation of the internal mammary chain. P-value <0.05 was considered to be statistical significant.

## Results

In total 524 patients were selected from the cancer registry, 3 patients were lost to follow up. Of the 521 patients evaluated, 180 were treated within the RT-group protocol. Three hundred and forty-one patients were treated with an ALND. In table 1 characteristics of both patient groups are shown. The difference in age between both groups was significant (p < 0.001). The median follow-up was 7.2 years (mean 7.7 years). The treatment characteristics are shown in table 2.

**Table 1:** patient and tumour characteristics

		RT-group	S-group
Number of patients		180	341
Age, years*		67	61
T stages (%)	pT1	67.8	79.5
	pT2	32.2	20.5
N stages (%)	PNx	100	-
	pN0	-	76.8
	pN1	-	22.9
	pN2	-	0.3

*p < 0.05

**Table 2:** treatment characteristics

	RT-group	S-group
RT axilla (%)	100%	5.9%
RT internal mammary chain (%)	78.3%	23.2%
Tamoxifen (%)	98.9%	22.3%
Chemotherapy (%)	0.6%	10.0%

After 5 years, the number of regional recurrences was very low and no differences were found between the 2 treatment groups, two (1.1%) in the RT-group and five (1.5%) in the S-group. In the RT-group one regional recurrence was found in the supraclavicular region and the other combined infraclavicular/axillar. In the S-group they were located in the supraclavicular region and the axilla in 2 cases each and one combined supraclavicular/axillar. After dissemination research two of these patients (1 in each group) showed distant metastasis.

The local control rates were better in the RT-group, with local recurrence rate of 2.2% and 3.5% at 5 years in the RT and the S-group respectively. Distant failures occurred less often in the RT-group: 6.7% vs.10.3%.

**Figure 1:**
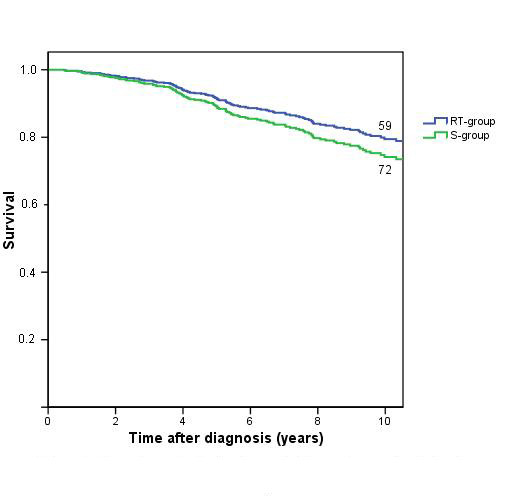
Overall survival by treatment, with numbers of patients at risk at 10 years.

The overall survival (OS) was similar in both groups. The 5 and 10-year OS rates in the RT-group were 92% and 80%, in the S group these were 90% and 75% respectively, figure 1.

**Figure 2:**
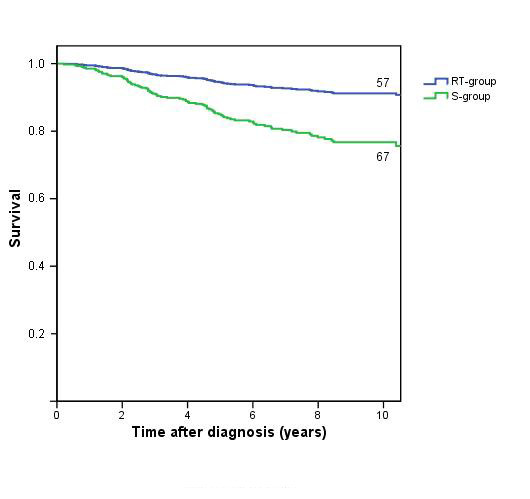
Disease free survival by treatment, with numbers of patients at risk at 10 years.

The disease free survival (DFS) was better in the RT-group, with a Hazard Ratio of 0.4 (95% CI: 0.3 – 0.8, p = 0.003) in the univariate analysis and 0.4 (95% CI: 0.2 – 0.7, p = 0.001) in the multivariate analysis corrected for age, stage and radiotherapy of the internal mammary chain, table 3; figure 2.

**Table 3:** results of univariate and multivariate analysis of disease free survival

		Univariate		Multivariate	
Factor	No	HR	95% CI	HR	95% CI

S-group	341	1	Reference	1	Reference
RT-group	180	0.4	0.3–0.8	0.4	0.2–0.7
RT internal mammary chain					
Yes	220	1	Reference	1	Reference
No	301	1.0	0.7–1.6	0.7	0.4–1.1
pT1	393	1	Reference	1	Reference
pT2	128	1.6	1.0–2.6	1.8	1.1–2.8
Age	521	1.0	0.9–1.0	1.0	0.9–1.0

## Discussion

The main issue of this article is the regional control in patients of 50 years and older with T1–2 cN0 breast cancer treated with breast conserving therapy and regional radiotherapy compared to an axillary lymph node dissection. The low regional recurrence rates in our study show that RT is a safe alternative for an ALND.

It is hard to draw firm conclusions in this non-randomized setting, where selection can not be ruled out, but some comparisons can be made.

Five studies with primary axillary radiotherapy have been published before [1,9,12-14]. Despite the somewhat different patient characteristics and adjuvant treatment they all confirm low regional recurrence rates. Compared to these articles we have a slightly better DFS, and a higher hormonal therapy usage, 98.9% vs. 2.4% – 71% [9,12,13]. They also conclude that RT equals an ALND with regard to survival, table 4.

**Table 4:** overview of comparable studies

	Patient characteristics				Results at 5 years			
	No	Age	HT(%)	CT(%)	RF(%)	DF(%)	DFS(%)	OS(%)

Spruit et al.	180	67	98.9	0.6	1.1	6.7	95	92
Louis-Sylvestre et al. [12]	332	51	2.4	2.7	2.2	12.8	82	94
Chua et al. [1]	229	64	13	2	2.1	Nm	Nm	Nm
Hoebers et al. [9]	105	64	71	0	0	8	82	83
Wong et al. [13] *	92	69	60	3	0	6	81	89
Wazer et al. [14]	73	74	90	0	0	9	84	70

* 3 years results

No number of patients

HT tamoxifen

RF regional failure

DF distant failure

DFS disease free survival

OS overall survival

Nm not mentioned in text

The disease free survival was significantly better in the RT-group, in the univariate as well as the multivariate analysis (p < 0.05). A few explanations are possible.

The number of patients irradiated on the internal mammary lymph nodes was significantly higher in the RT-group. We cannot tell what the influence of this on the DFS has been. Probably the results of the EORTC trial, [11] will clarify this.

Locoregional radiotherapy, which was given to all the patients in the RT-group, gives a better locoregional control and even a better survival in high risk postmenopausal breast cancer patients [15]. Recently Overgaard described in a subanalysis of the DBCG 82 b&c trial that intermediate risk patients (1–3 positive lymph nodes) benefit from locoregional radiotherapy, not only for locoregional control but also for survival [16]. It can be assumed that the percentage of positive axillary lymph nodes would be similar in both groups. Therefore it is very well possible that the RT-group contains patients (23%) that would have been classified as intermediate risk breast cancer. Whether this explains the better DFS in our study group is hard to tell. In October 2006 an EORTC study started to give answers on that matter too (SUPREMO) [17].

On the other hand, there are some reports showing that no treatment of the axilla at all in early breast cancer, is an option too [18-20]. These studies treated patients with higher age (>70 yr) and/or smaller tumours (< 1.2 cm) than we did. The tamoxifen usage was at least 90% in all of them. The axillary recurrences rates in these studies varied from 1,5% to 5.4%.

Tamoxifen was prescribed to all patients in the RT-group. Literature shows that tamoxifen not only diminishes the amount of distant failures but the local recurrences as well [21]. It could be that this explains the differences in local and distant failures between our two groups.

As mentioned before no reliable evaluation of the complications like lymph oedema or shoulder impairment could be made.

The last few years the sentinel node procedure seems to have proven its value. In the Netherlands staging of the axilla in low risk breast cancer is routinely performed by the SNP. Because the sentinel node biopsy is less invasive, it gives less morbidity than an ALND. If the sentinel node is tumour negative no further treatment of the axilla is necessary. If the sentinel node shows metastases the axilla needs to be treated, either with an ALND or with radiotherapy [22]. The combination of a SNP and axillary radiotherapy will probably not lead to substantial morbidity, as we see after an ALND in combination with radiotherapy of the axilla.

It is to be expected that in the future an ALND will be of lesser importance in the staging of breast carcinoma, especially with the upcoming techniques like Her2-neu expression and genomics. Together with the findings in the sentinel node the indication for adjuvant therapy will be determined. A randomized multicentre trial is investigating the difference between an ALND and axillary, supra- and infraclaviculary radiotherapy in patients with a positive sentinel node biopsy (AMAROS trial) [23].

## Conclusion

Regional recurrence rates after regional radiotherapy are very low and can safely replace surgery in a clinically negative axilla.

## Competing interests

The author(s) declare that they have no competing interests.

## Authors' contributions

PS: collected data, wrote the article

SS: conducted data evaluation, statistical analysis, critical review of the manuscript

ME: conducted data evaluation, statistical analysis

EV: collected data, critical review of the manuscript.

CH: designed the RT-group treatment protocol, collected data, critical review of the manuscript.

All authors read and approved the final manuscript.
